# Emergence of cryptic species and clades of *Meyerozyma guilliermondii* species complex exhibiting limited *in vitro* susceptibility to antifungals in patients with candidemia

**DOI:** 10.1128/spectrum.05115-22

**Published:** 2023-09-12

**Authors:** Elaine Cristina Francisco, Felipe de Camargo Ribeiro, João Nobrega Almeida Junior, Diego Betto Pedoni, Daniel Archimedes da Matta, Maribel Dolande, Analy Salles de Azevedo Melo, Ricardo Ferreira Lima, Valério Rodrigues Aquino, Dora E Corzo-León, Jeannete Zurita, Jorge Alberto Cortes, Marcio Nucci, Arnaldo Lopes Colombo

**Affiliations:** 1 Laboratório Especial de Micologia, Disciplina de Infectologia, Universidade Federal de São Paulo, São Paulo, Brazil; 2 Department of Mycology, Instituto Nacional de Higiene Rafael Rangel, Caracas, Venezuela; 3 Hospital de Clínicas, Universidade Federal do Rio, Porto Alegre, Grande do Sul, Brazil; 4 MRC Centre for Medical Mycology, University of Exeter, Exeter, United Kingdom; 5 Unidad de Investigaciones en Biomedicina, Zurita & Zurita Laboratorios, Quito, Ecuador; 6 Facultad de Medicina, Pontificia Universidad Catolica del Ecuador, Quito, Ecuador; 7 Facultad de Medicina, Universidad Nacional de Colombia, Sede Bogotá, Bogotá, Colombia; 8 Departamento de Clínica Médica, Universidade Federal do Rio de Janeiro, Rio de Janeiro, Brazil; Universidade de Brasilia, Brasilia, Brazil

**Keywords:** *Meyerozyma guilliermondii *species complex, candidemia, antifungal resistance, nosocomial infection, emerging yeast pathogen

## Abstract

Members of the *Meyerozyma guilliermondii* species complex are able to cause superficial and life-threatening systemic infections with low susceptibility to azoles and echinocandins. We tested 130 bloodstream *M. guilliermondii* complex isolates collected from eight Latin American medical centers over 18 years (period 1 = 2000–2008 and period 2 = 2009–2018) to investigate trends in species distribution and antifungal resistance. The isolates were identified by rDNA ITS region sequencing, and antifungal susceptibility tests were performed against fluconazole, voriconazole, anidulafungin, and amphotericin B using the CLSI microbroth method. *M. guilliermondii sensu stricto* (s.s.; *n* = 116) was the most prevalent species, followed by *Meyerozyma caribbica* (*n* = 12) and *Meyerozyma carpophila* (*n* = 2). Based on rDNA ITS identification, three clades within *M. guilliermondii sensu stricto* were characterized (clade 1 *n* = 94; clade 2 *n* = 19; and clade 3 *n* = 3). In the second period of study, we found a substantial increment in the isolation of *M. caribbica* (3.4% versus 13.8%; *P* = 0.06) and clade 2 *M. guilliermondii* s.s. exhibiting lower susceptibility to one or more triazoles.

**IMPORTANCE:**

Yeast-invasive infections play a relevant role in human health, and there is a concern with the emergence of non-*Candida* pathogens causing disease worldwide. There is a lack of studies addressing the prevalence and antifungal susceptibility of different species within the *M. guilliermondii* complex that cause invasive infections. We evaluated 130 episodes of *M. guilliermondii* species complex candidemia documented in eight medical centers over 18 years. We detected the emergence of less common species within the *Meyerozyma* complex causing candidemia and described a new clade of *M. guilliermondii* with limited susceptibility to triazoles. These results support the relevance of continued global surveillance efforts to early detect, characterize, and report emergent fungal pathogens exhibiting limited susceptibility to antifungals.

## INTRODUCTION

Yeasts of the *Meyerozyma guilliermondii* species complex are widely distributed in nature, being isolated from insect-related sources, aquatic habitats, soil, amphibians, birds, maize roots from semiarid regions, and humans (1 – 5). Based on the current taxonomy, the genetic heterogeneous *Meyerozyma guilliermondii* species complex includes *M. guilliermondii sensu stricto* (s.s.; *Pichia guilliermondii*), *M. caribbica* (*P. fermentati*), *M. carpophila* (*Candida guilliermondii* var. *carpophila*), *M. smithsonii, M. athensensis*, *M. elateridarum*, *M. amylolytica,* and *M. neutonensis* (1, 6).

*M. guilliermondii* species complex is able to cause superficial and invasive infections in humans. One to 12% of all *Candida* spp. causing nosocomial bloodstream infections may be related to the *M. guilliermondii* species complex, and higher prevalence rates have been reported in Canada, Latin American, and Asian medical centers (3, 7 – 12). Specifically in cancer patients, the prevalence of this particular pathogen may range between 26% and 41% (13, 14).

Data collected from medical institutions in Argentina, Taiwan, and China suggested that *M. guilliermondii* s.s. was responsible for 70–95% of all episodes of candidemia caused by this species complex, followed by *M. caribbica* and *M. carpophila* (15 – 20). However, most of these studies reported experiences from single centers, where a limited number of isolates cultured from patients with invasive infections have been accurately identified by molecular methods and checked for *in vitro* antifungal susceptibility. As far as we know, there is only one multicenter study addressing the frequency of *M. guilliermondii* species complex causing systemic infection in hospitalized patients (20).

The present study evaluated the prevalence rate of cryptic species within the *M. guilliermondii* species complex causing fungemia in patients admitted to eight medical centers from Latin American countries, providing data on their *in vitro* susceptibility profile against four antifungal agents licensed to treat patients with candidemia.

## MATERIALS AND METHODS

### Selection of clinical isolates

We included 130 *M*. *guilliermondii* species complex isolates obtained from patients with candidemia diagnosed between 2000 and 2018 in eight Latin American medical centers located in seven countries. Isolates presumptively identified as *M. guilliermondii* (syn. *C. guilliermondii*) by conventional biochemical methods were sent to the Special Laboratory of Mycology, Universidade Federal de São Paulo, Brazil, for further accurate species identification and antifungal susceptibility testing (AST). The isolates selected for the present study were all cultured as part of epidemiologic multicenter studies coordinated by our group where all investigators were requested to systematically collect cases from episodes of candidemia sequentially documented in their hospitals during the study period (8, 21 – 24).

The following reference strains were added as quality control*: M. guilliermondii* (ATCC6260)*, M. caribbica* (CBS9966), *C. membranifaciens* (CBS1952), *C. krusei* (ATCC6258), and *C. parapsilosis* (ATCC22019). All isolates were stored at −80°C in liquid YEPD medium plus 20% glycerol.

### Molecular identification

Molecular identification was performed by sequencing the ITS region of rDNA using the V9G (5′-TTACGTCCCTGCCCTTTGTA-3′) and LS266 (5′-GCATTCCCAAACAACTCGACTC-3′) primer pairs as previously described (24). Raw sequences were assembled based on two to four reads per isolate and edited using Phred-Phrap-Consed targeting a Phred score >30 (25).

The sequences were aligned by muscle algorithm and manually corrected using MEGA X software (26). The final species identification and the intraspecific diversity investigation were carried out by phylogenetic analyses using the Neighbor-Joining method based on the Kimura two-parameter model with 1,000 bootstrap pseudo-replicates considering gap positions (25 – 27). Type strain sequences of *M. guilliermondii* species complex and other *Candida* species deposited in the GenBank (https://www.ncbi.nlm.nih.gov/genbank/) were included in the phylogenetic analysis.

The phylogenetic tree was created and edited with Fig Tree software version 1.4.4 (http://tree.bio.ed.ac.uk/software/figtree/).

### *In vitro* antifungal susceptibility testing

AST was performed according to CLSI M27ed4^th^ broth microdilution method (28). The following antifungal agents were tested: fluconazole (FLC), voriconazole (VRC), anidulafungin (AFG), and amphotericin B (AMB). Minimal inhibitory concentrations (MICs) were determined by visual readings after 24 h of incubation based on the lower concentration able to inhibit 50% of cell growth for azoles and AFG and 100% growth inhibition for AMB. The 97.5% epidemiologic cutoff (ECV) values were used to classify the isolates as wild type or non-wild type according to Espinel-Ingroff and Turnidge (29) and Espinel-Ingroff et al. (30).

### Statistical analysis

In order to characterize historical trends in terms of species distribution within the *M. guilliermondii* species and their antifungal susceptibility, we arbitrarily divided the study period in 2 intervals: period 1 from 2000 to 2008 with 58 isolates of *M. guilliermondii* species complex and period 2 from 2008 to 2018 with 72 *M*. *guilliermondii* species complex isolates. Prevalence rates of different species (period 1 versus period 2) were compared by χ2 tests. Antifungal MIC values obtained in both periods as well as the prevalence rates of isolates exhibiting results above the ECV values expected for *M. guilliermondii* (30) were compared by a Kruskal–Wallis test. Finally, we compared the MICs of FLC, VRC, AMB, and AFG generated by different *M. guilliermondii* clades by using the Student’s *t*-test. All tests with *P* < 0.05 were considered statistically significant.

## RESULTS

### Molecular species identification

A total of 130 *M*. *guilliermondii* species complex isolates were collected including 81 from Brazil (62.3%), 27 from Honduras (20.8%), 9 from Argentina (7%), 8 from Venezuela (6%), 3 from Colombia (2.3%), and 1 each from Ecuador (0.8%) and Mexico (0.8%).

**Fig 1:**
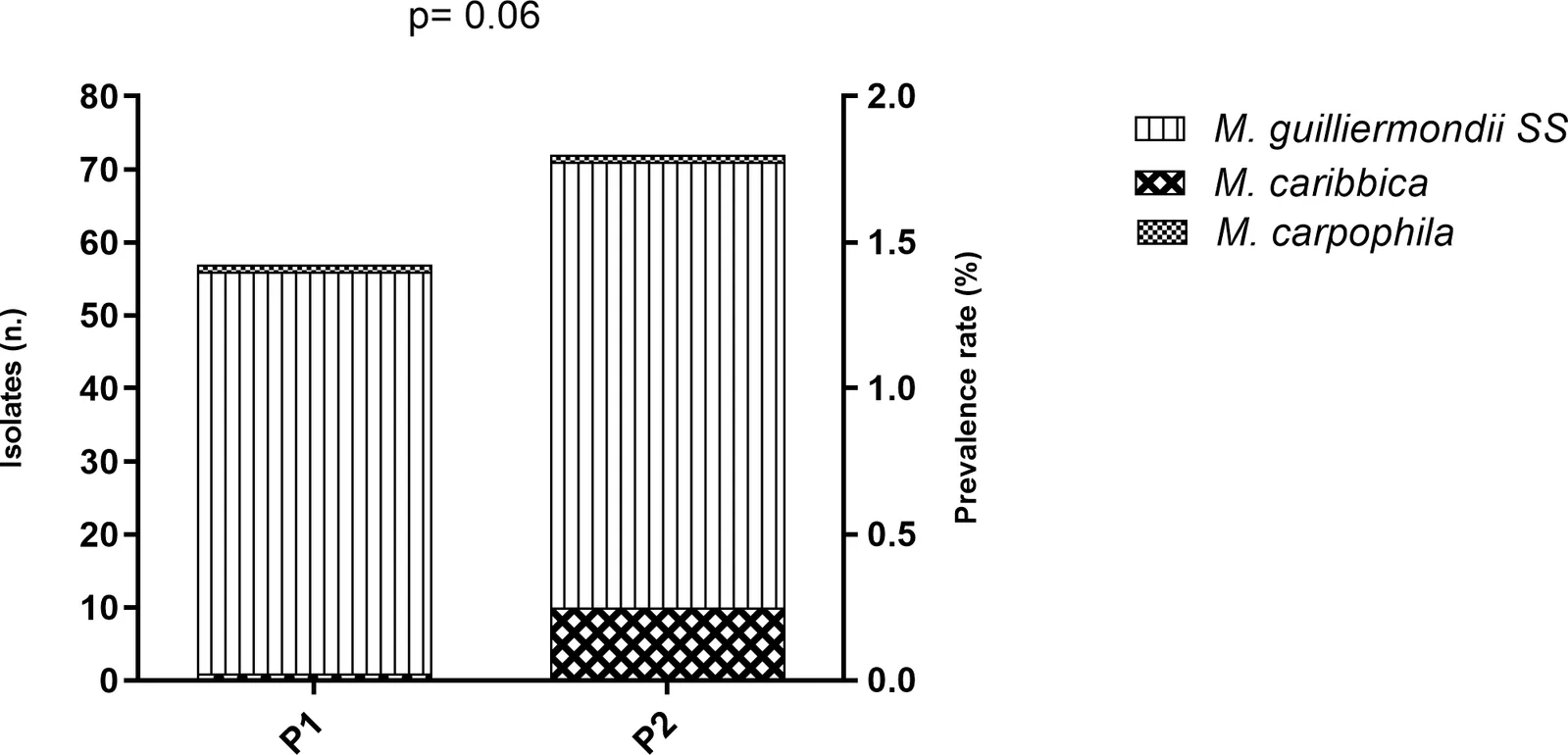
Prevalence rates of different species within the *Meyerozyma guilliermondii* complex documented along the study; Period 1 (P1) comprising 58 isolates collected during 2000–2008; Period 2 (P2) comprising 72 isolates collected during 2009–2018; *n*., number of isolates; s.s., *sensu stricto*.

*Meyerozyma guilliermondii* s.s. was the most frequent species found (*n* = 116; 89.2%), followed by *M. caribbica* (*n* = 12; 9.2%) and *M. carpophila* (*n* = 2; 1.6%). Comparing the two periods, we observed a non-significant decrease in the percentage of *M. guilliermondii* s.s., from 94.8% in period 1 to 84.7% in period 2 (*P* = 0.66). On the other hand, there was an increase in the proportion of *M. caribbica* during period 2 (13.8% versus 3.4% in period 1, *P* = 0.06) (Fig. 1). Of note, *M. caribbica* was found in Ecuador (*n* = 1), Venezuela (*n* = 3), and in four different Brazilian medical centers located in São Paulo city (*n* = 8). The two isolates of *M. carpophila* were recovered from two different Brazilian hospitals in 2003 and 2010.

**Fig 2:**
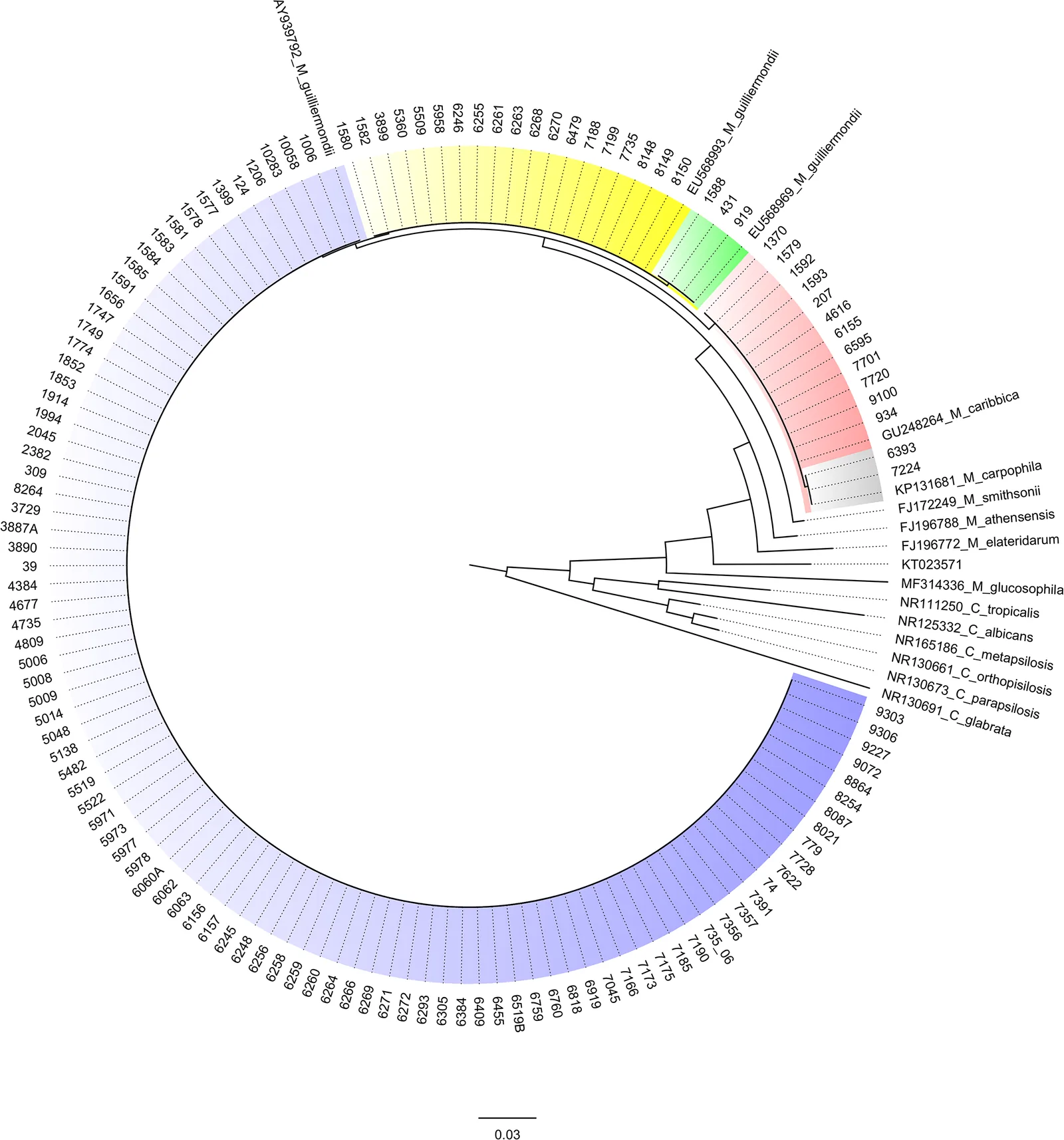
Neighbor-joining polar tree of *M. guilliermondii* species complex isolates and *Candida* species based on ITS rDNA sequences. Sequences of non-*M*. *guilliermondii* species complex were obtained from GenBank (accession number = NR111250; NR125332; NR165186; NR130661; NR130673; NR130691).

As shown in Fig. 2 and Fig. S1, after conducting a neighbor-joining analysis, we characterized three different clades within *M. guilliermondii* s.s. isolates: (i) clade 1 aligned 94 clinical isolates and GenBank sequence AY939792; (ii) clade 2 aligned 19 clinical isolates and GenBank sequence EU568993; (iii) clade 3 aligned three clinical isolates and GenBank sequence EU568969. Of note, a substantial increase in the prevalence of *M. guilliermondii* clade 2 was found in period 2 (*n* = 26 isolates versus *n* = 3 isolates in period 1; *P* = 0.001).

### Antifungal susceptibility testing

Table 1 summarizes the antifungal susceptibility results for *M. guilliermondii* s.s. and *M. caribbica* isolates. Due to the unexpressed number of *M. carpophila* isolates tested, we were not able to include this species in our statistical analysis. The two *M. carpophila* isolates presented the following MIC results: 1 µg/mL for FLC, 0.03 µg/mL for VRC, and 0.5 µg/mL for AFG and AMB.

**TABLE 1:** Antifungal susceptibility tests of 128 *Meyerozyma guilliermondii* complex isolates against fluconazole, voriconazole, anidulafungin, and amphotericin B (CLSI microbroth dilution)

		Period 1: 2000–2008					Period 2: 2009–2018				
Species	Antifungal (µg/mL)	MIC_50_	MIC_90_	GM	Range	% >ECV	MIC_50_ ^*a*^	MIC_90_	GM	Range	% >ECOFF
*M. guilliermondii* (*n* = 116)	Fluconazole	2	16	2.30	1–16	0%	2	32	3.04	1–32	6.55%
	Voriconazole	0.06	0.25	0.04	0.03–0.05	3.63%	0.06	1	0.12	0.03–1	11.47%
	Anidulafungin	2	4	2	0.125–4	-^*b*^	1	4	1.58	0.05–4	-
	Amphotericin B	0.25	1	0.5	0.03–0.04	-	0.25	0.5	0.33	0.06–1	-
*M. caribbica* (*n* = 12)	Fluconazole	2	4	2.83	2–4	0%	4	8	4.28	1–16	10%
	Voriconazole	0.03	0.03	0.03	0.03	0	0.06	0.25	0.03	0.03–0.25	30%
	Anidulafungin	1	2	1.41	1–2	-	2	4	2.29	2–4	-
	Amphotericin B	0.25	0.5	0.86	0.25–0.5	-	0.25	1	0.57	0.25–1	-

^*a*^ MIC_50_, minimum inhibitory concentration able to inhibit 50% of all isolates tested; MIC_90_, minimum inhibitory concentration able to inhibit 90% of all isolates tested; GM, geometric mean; %>ECV, percentage of isolates with MICs above the epidemiological cut-off values; *n*., number of isolates tested.

^*b*^ "-": ECV not available for the particular drug and species tested.

Table 2 summarizes antifungal MIC ranges, geometric means, MIC_50_, MIC_90_ values, and percentage of isolates exhibiting MIC results above the ECV values for clades 1 (*n* = 94) and 2 (*n* = 19) of *M. guilliermondii* s.s. isolates. The clade 2 isolates exhibited higher GM MIC values for FLC and VRC (GM = 5.97 µg/mL and 0.16 µg/mL, respectively, *P* = 0.008) when compared to clade 1 isolates (GM = 2.35 µg/mL and 0.05 µg/mL, *P* = 0.006). We failed to identify any significant differences in AFG GM MIC values among isolates representative of clade 1 and clade 2 (GM = 1.68 µg/mL and 1.2 µg/mL, respectively, *P* = 0.53). The AMB GM MIC values were 0.28 µg/mL and 0.33 µg/mL for clade 1 and clade 2 isolates, respectively (*P* = 0.44). The three clade 3 isolates tested presented MIC values of 2 µg/mL for FLC, 0.03–0.06 µg/mL for VRC, 2 µg/mL for AFG, and 0.25–0.5 µg/mL for AMB.

**TABLE 2:** Antifungal susceptibility tests of 116 *Meyerozyma guilliermondii sensu stricto* (clades 1 and 2) against fluconazole, voriconazole, anidulafungin, and amphotericin B (CLSI microbroth dilution)

Clades		Fluconazole (µg/mL)	Voriconazole (µg/mL)	Anidulafungin (µg/mL)	Amphotericin B (µg/mL)
Clade 1 (*n* = 94)	MIC_50_ ^*a*^	2	0.06	2	0.25
	MIC_90_	16	0.5	4	1
	Range	1–32	0.03–0.5	0.125–4	0.06–1
	GM	2.35	0.05	1.68	0.28
	% >ECV	1.06%	2.17%	−	−
Clade 2 (*n* = 19)	MIC_50_	4	0.06	1	0.25
	MIC_90_	32	1	2	0.5
	Range	1–32	0.03–1	0.5–2	0.125–0.5
	GM	5.97	0.16	1.2	0.33
	% >ECV	21.05%	42.10%	−	−

^*a*^ MIC_50_: minimum inhibitory concentration able to inhibit 50% of all isolates tested; MIC_90_, minimum inhibitory concentration able to inhibit 90% of all isolates tested; GM, geometric mean; %>ECV, percentage of isolates with MICs above the epidemiological cut-off values; (−), non-applicable; *n*, number of isolates.

The percentage of *M. guilliermondi* s.s. isolates exhibiting MIC results above ECV values of FLC and VRC increased from 0% in period 1 to 6.55% in period 2 (*n* = 0 versus *n* = 4; *P* = 0.02), and from 3.63% in period 1 to 11.47% in period 2 (*n* = 2 versus *n* = 7; *P* = 0.01), respectively. This finding was correlated with a rise in incidence of clade 2 *M*. *guilliermondii* s.s. isolates in period 2 (*n* = 3 versus *n* = 16; *P* = 0.01; see Table 2). Isolates of *M. caribbica* were more likely to be documented among episodes of candidemia occurring in period 2 (*n* = 2; 3.4% versus *n* = 10; 13.8%, *P* = 0.06). In addition, the percentage of *M. caribbica* isolates exhibiting MICs above the ECV value for VRC was also higher in period 2 (*n* = 0 versus *n* = 3, 30%; *P* = 0.04).

All isolates of *M. guilliermondii* tested were considered wild type to amphotericin B regardless of the species tested and period of evaluation. Regarding AFG, all isolates of *M. guilliermondii* tested showed MIC results below to ECV values.

## DISCUSSION

Despite being considered an uncommon cause of candidemia, reports from medical centers worldwide suggest that *M. guilliermondii* species complex may be especially prevalent in patients with cancer, neutropenia, and critically ill individuals submitted to surgical procedures (7, 8, 13 – 17).

In the present study, *M. guilliermondii* s.s. was the most common species found (*n* = 116; 89.2%), followed by *M. caribbica* (*n* = 12; 9.2%) and *M. carpophila* (*n* = 2; 1.6%). Our findings are similar to data reported in other single center studies published from Argentina, Spain, Taiwan, and China where *M. guilliermondii* s.s. was more prevalent than other species within this complex (69% to 95.5% of all isolates). According to these studies, the prevalence of *M. caribbica* ranges from 4.5% to 22.7% (16, 17, 19, 20). Finally, *M. carpophila* has been rarely reported as a cause of superficial or invasive infection in humans globally. So far, we found only two reports of clinical isolates of *M. carpophila* causing infection, one from Argentina (19) and other from Iran (31).

Despite being less prevalent than *M. guilliermondii* s.s., there was a trend for increasing rates of *M. caribbica* during period 2, as shown in Fig. 1. Indeed, a recent retrospective study performed in Brazil by Chaves et al. (32) evaluating isolates collected between 2014 and 2016 showed that five out 10 *M*. *guilliermondii* species complex isolates obtained from patients with candidemia were identified as *M. caribbica* (the other five were identified as *M. guilliermondii* s.s.). An increased rate of candidemia caused by *M. caribbica* was also reported in a Spanish study (17).

Cryptic species of *M. guilliermondii* may exhibit characteristics in their antifungal susceptibility profile. In this regard, our 12 *M*. *caribbica* isolates tested presented higher MICs for FLC, VRC, and AFG when compared to *M. guilliermondii* s.s. Other authors have also described these findings (32, 33). By contrast, Marcos-Zambrano et al. (17) did not find any difference in the susceptibility profile between *M. caribbica* and *M. guilliermondii* s.s. (17). Further studies evaluating a larger number of clinical isolates are necessary to better understand the potential biological characteristics of *M. guilliermondii* and *M. caribbica* isolates.

Considering that most guidelines suggest the use of echinocandins as first-line treatment for candidemia, caution and additional vigilance should be taken to monitor patients with candidemia caused by *M. guilliermondii* species complex. This species complex exhibits natural *FKS1* gene mutations L633M and I634A that reduce susceptibility to echinocandins (34). Marcos-Zambrano et al. (17) reported that persistent candidemia (positive blood cultures >2 d after treatment initiation) was seen more frequently in infections by *M. guilliermondii* species complex than by *C. albicans*.

To correctly identify the cryptic species of *M. guilliermondii* complex, we used the ITS region as previously proposed (35). Besides accurate molecular identification of *Candida* at species level, the rDNA ITS region sequencing data may support the characterization of intraspecific genetic diversities among *M. guilliermondii* s.s. isolates. Other authors have described the presence of genetic heterogeneity among *M. guilliermondii* s.s. isolates collected from different geographic regions and biological materials (24, 36). Corte et al. (36) found physiological and genetic differences among 57 *M*. *guilliermondii* isolates collected from environment and clinical sources. Based on ITS sequence findings, the authors separated fruit strains from clinical samples that grouped in two different branches by using neighbor-joining analysis. Merseguel et al. (24) also found a genetic diversity among 22 *M*. *guilliermondii* clinical isolates characterized by the same DNA marker. Despite characterizing genetic diversity within *M. guilliermondii* s.s. isolates, the authors did not explore any differences of these clades in terms of antifungal susceptibility.

In the present study, after checking for intraspecific genetic diversity by using neighbor-joining analysis of ITS sequencing, we were able to identify three different clades (clade 1, clade 2, and clade 3) circulating in Latin America. By checking historical trends in the prevalence of clades among *M. guilliermondii* s.s. isolates we demonstrated an increased rate of infections caused by clade 2 during the second period of study. It is noteworthy that clade 2 isolates were less susceptible to FLC and VRC when compared to other clades (see Table 2).

Limitations of our study include the lack of clinical information about the episodes of fungemia caused by *M. guilliermondii* s.s., precluding any discussion about the correlation between *in vitro* data and clinical response to therapy, as well as the identification of clinical variables that could explain the emergence of *M. caribbica* in the second period of our survey.

In conclusion, our main findings were: (i) *M. guilliermondii* s.s. was the most common species isolated in cases of *M. guilliermondii* candidemia diagnosed in eight Latin American medical centers, followed by *M. caribbica* and *M. carpophila*; (ii) by using neighbor-joining analysis of ITS sequencing data, we documented three different clades among the 116 *M*. *guilliermondii* s.s. clinical isolates tested (clade 1, clade 2, clade 3); (iii) in the second period of study (P2, 2009–2018) we found a substantial increase in the isolation of *M. caribbica* and clade 2 *M. guilliermondii* s.s. exhibiting lower susceptibility against both azoles tested. The emergence of less common species and clades of *M. guilliermondii* with limited susceptibility to antifungal drugs are of great concern and support the relevance of continued global surveillance efforts to detect, characterize, and report emergent fungal pathogens exhibiting limited susceptibility to antifungals.

## Data Availability

The sequence data have been deposited at the GenBank Sequence database under accession numbers MT524274–MT525130 (Table S1).

## SUPPLEMENTAL MATERIAL

Figure S01 spectrum.05115-22-s0001.pdf Phylogenetic tree, including figure and caption.

Table S1 spectrum.05115-22-s0002.xlsx Accession numbers.

ASM does not own the copyrights to Supplemental Material that may be linked to, or accessed through, an article. The authors have granted ASM a non-exclusive, world-wide license to publish the Supplemental Material files. Please contact the corresponding author directly for reuse.
