# Supplementary material for: Emergence of cryptic species and clades of Meyerozyma guilliermondii species complex exhibiting limited in vitro susceptibility to antifungals in patients with candidemia
Source: Microbiol Spectr. 2023 Sep 12;11(5):e05115-22. doi: 10.1128/spectrum.05115-22 (PMC10580822; doi:10.1128/spectrum.05115-22)
Supplement: Figure S01 — Phylogenetic tree, including figure and caption. [file spectrum.05115-22-s0001.pdf]

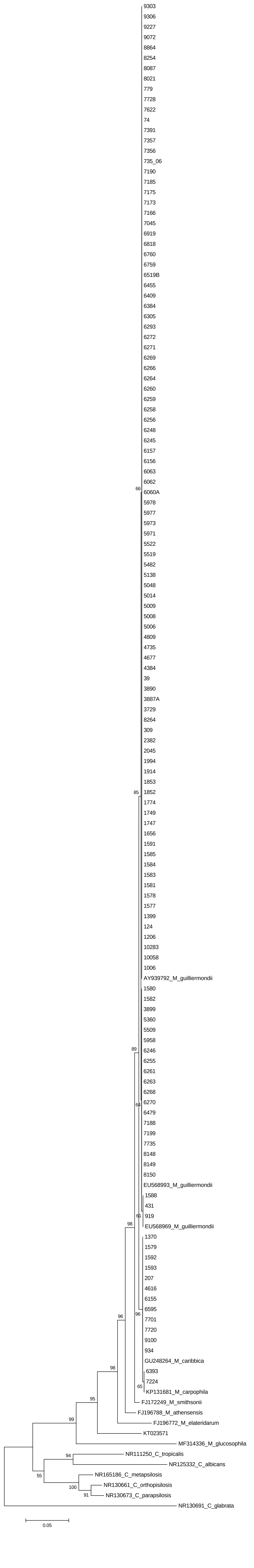
9303
9306
9227
9072
8864
8254
8087
8021
779
7728
7622
74
7391
7357
7356
735_06
7190
7185
7175
7173
7166
7045
6919
6818
6760
6759
6519B
6455
6409
6384
6305
6293
6272
6271
6269
6266
6264
6260
6259
6258
6256
6248
6245
6157
6156
6063
6062
66
6060A
5978
5977
5973
5971
5522
5519
5482
5138
5048
5014
5009
5008
5006
4809
4735
4677
4384
39
3890
3887A
3729
8264
309
2382
2045
1994
1914
1853
85
1852
1774
1749
1747
1656
1591
1585
1584
1583
1581
1578
1577
1399
124
1206
10283
10058
1006
AY939792_M_guilliermondii
1580
1582
3899
5360
5509
5958
89
6246
6255
6261
6263
6268
64
6270
6479
7188
7199
7735
8148
8149
8150
EU568993_M_guilliermondii
1588
431
61
919
98
EU568969_M_guilliermondii
1370
1579
1592
1593
207
4616
6155
6595
96
96
7701
7720
9100
934
GU248264_M_caribbica
6393
7224
98
65
KP131681_M_carpophila
95
FJ172249_M_smithsonii
FJ196788_M_athensensis
99
FJ196772_M_elateridarum
KT023571
MF314336_M_glucosophila
94
NR111250_C_tropicalis
NR125332_C_albicans
55
NR165186_C_metapsilosis
100
NR130661_C_orthopsilosis
91
NR130673_C_parapsilosis
NR130691_C_glabrata
0.05

Caption

Figure S01. Neighbor-joining tree of *M. guilliermondii* species complex isolates and *Candida* species based on ITS rDNA sequences. Reference sequences of *M. guilliermondii* and non-*M. guilliermondii* species complex were obtained from GenBank (accession numbers= *M. glucosophila* MF314336; *M. elateridarum* FJ196772; *M. carpophila* P131681; *M. smithsonii* FJ172249; *M. athensensis* FJ196788; *M. caribbica* GU248264; *M. guilliermondii* EU568969; *M. guilliermondii* EU568993; *M. guilliermondii* AY939792; *Candida tropicalis* NR111250; *Candida albicans* NR125332; *Candida metapsilosis* NR165186, *Candida orthopsilosis* NR130661; *Candida parapsilosis* NR130673; and [*Candida*] *glabrata* NR130691).
